# Abundance of *Ixodes ricinus* Ticks (Acari: Ixodidae) and the Diversity of *Borrelia* Species in Northeastern Poland

**DOI:** 10.3390/ijerph19127378

**Published:** 2022-06-16

**Authors:** Katarzyna Kubiak, Hanna Szymańska, Małgorzata Dmitryjuk, Ewa Dzika

**Affiliations:** 1 Department of Medical Biology, Collegium Medicum, School of Public Health, University of Warmia and Mazury in Olsztyn, Zolnierska 14c, 10-561 Olsztyn, Poland; hanna.szymanska@uwm.edu.pl (H.S.); e.dzika@uwm.edu.pl (E.D.); 2 Department of Biochemistry, Faculty of Biology and Biotechnology, University of Warmia and Mazury in Olsztyn, Oczapowskiego 1A, 10-719 Olsztyn, Poland; m.dmit@uwm.edu.pl

**Keywords:** questing ticks, density, *Borrelia burgdorferi* sensu lato complex, *Borrelia* species co-infections

## Abstract

Monitoring the abundance of ticks and the prevalence of pathogens in ticks is an important activity in assessing the risk of tick-borne diseases and helps to develop preventive measures. This study aimed to estimate the density of *Ixodes ricinus*, the prevalence of *Borrelia* species, and their diversity in northeastern Poland. The overall mean *I. ricinus* density was 9.7 ticks/100 m^2^. There were no differences between years, subregions, or habitats of study. The *Borrelia* infection rate was higher in females (22.6%) and males (14.3%) than in nymphs 5.5% (MIR). The most infected ticks came from the eastern subregion (10.1%) where the incidence of borreliosis among the inhabitants was over 20% higher than in the other subregions. In the infected ticks, *B. afzelii* (38.3%) and *B. garinii* (34.5%) were predominant. *B. bavariensis* was confirmed in *I. ricinus* in Poland for the first time. The most polymorphic was *B. garinii*. *B. miyamotoi* (belonged to the European type) was identified as a mono-infection in 0.9% of ticks and in 1.5% as a co-infection with *B. afzelii* and with *B. garinii*. Besides the risk of borreliosis and co-infections with different *Borrelia* species, physicians should also be aware of *B. miyamotoi* infections among patients.

## 1. Introduction

Significant ecological changes in climate and habitat caused by human population growth, such as urbanisation and agricultural intensification, contribute to the increase in the (re-) emergence of infectious diseases (EIDs) [1,2]. According to the World Health Organization (WHO), 75% of emerging infectious diseases that have affected people over the past three decades have originated from animals [3]. Among zoonosis, 22% of them are vector-borne and transmitted mainly by mosquitoes and ticks [4,5]. The most commonly diagnosed disease transmitted to humans by ticks of the genus *Ixodes* in the northern hemisphere, including the United States and Europe [6,7], is Lyme borreliosis (LB). LB is considered the prototype of a tick-borne emerging infectious disease [7]. Since 1983, when the association of infection with *Borrelia* spirochetes with clinical symptoms in humans was first documented, the worldwide burden of LB has increased and extended into regions and countries where the disease was not previously reported [8,9]. In Europe, the number of cases has increased steadily, with more than 360,000 cases reported over the last two decades. Every year over 85,000 LB cases are recorded, with the highest incidence in Central Europe (Czech Republic, Estonia, Lithuania, Slovakia, and Poland) [10,11].

The etiological agents of LB are the spirochetes of the *Borrelia burgdorferi* sensu lato (s.l.) complex, which is comprised of 21 species [12]. Six of them (*B. burgdorferi* sensu stricto (s.s.), *B. afzelii*, *B. garinii*, *B. bavariensis*, *B. lusitaniae*, and *B. spielmanii*) are regarded as human pathogens [7,12]. However, the DNA of *B. valaisiana* and *B. bissettii* has also been detected in tissues of symptomatic patients, so the pathogenicity of these species is not excluded, but still unclear [13,14,15,16,17]. The participation of *Ixodes* ticks was also confirmed in the transmission of *B. miyamotoi*, a species closely related to LB spirochetes, which is included in the tick-borne relapsing fever (TBRF) group [18,19,20,21]. Since 2011, when the first symptomatic case of human infection was recorded in Russia, *B. miyamotoi* has been classified as a human pathogen [22]. Further cases of human infection were reported in the USA [23,24], Europe (the Netherlands, Germany, Poland, Sweden, and France) [25,26,27,28], and Asia [29,30,31].

The genetic differentiation of *Borrelia* spirochetes at the inter- and intraspecies levels is associated with a wide range of non-specific clinical symptoms, making infection difficult to diagnose, treat, and produce effective preparations for immunoprophylaxis [8,32]. There were also differences noted in the geographical distribution of individual *Borrelia* species and changes in their prevalence over time [11,33], which is directly influenced by the occurrence of vectors (ticks) and reservoirs (vertebrates) of *Borrelia* spp. spirochetes. This depends on the conditions in local ecosystems and global environmental and socio-economic changes resulting from human activity [34]. Therefore, monitoring the distribution of ticks in the environment and tick infection rate with pathogenic, non-pathogenic/conditionally pathogenic, or new species (e.g., *B. miyamotoi*) is one of the main activities in assessing the risk of tick-borne diseases [33,35]. It is also an important contribution to understanding LB epidemiology and helps to diagnose and develop preventive measures.

**Figure 1:**
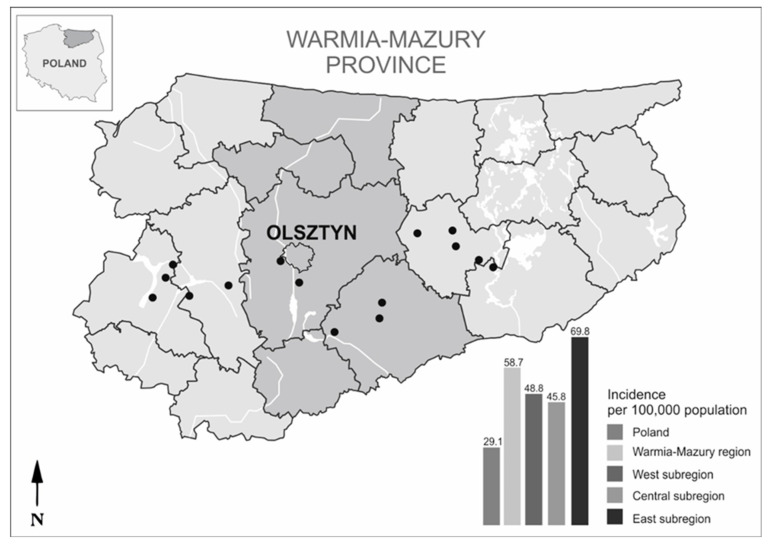
Tick collection sites located in western, central, and eastern subregions of northeastern Poland. Bars show the mean incidence of Lyme borreliosis in subregions, the Warmia and Mazury region, and in Poland for 2010–2015 (data obtained from the annual reports of selected infectious diseases (document MZ-57) registered in the Warmia and Mazury province by the Department of Epidemiology of the Voivodeship Sanitary-Epidemiological Station in Olsztyn and listed in the annual reports of the NIPH-NIH, Poland). The map was designed in CorelDRAWX5 based on Google Maps (https://www.google.pl/maps, accessed on 20 May 2020).

Northeastern Poland (the Warmia and Mazury region) is particularly rich in habitats that are favourable to ticks and reservoir species for *Borrelia* spp. This region could be classified as an LB “hotspot” since, among the inhabitants of this region, the incidence of LB is nearly two times greater than in the rest of Poland [36] (Figure 1). Despite this fact, the data on the diversity and prevalence of *Borrelia* species in *I. ricinus* in northeastern Poland are historical and, due to the methods used, they concern only three pathogenic *Borrelia* species (*B. burgdorferi* s.s., *B. garinii*, and *B. afzelii*) [37,38] and do not cover the entire region [39,40,41].

The aims of the study were: (1) to assess the tick density and infection rate of *Borrelia* spirochetes in the population of *I. ricinus* ticks in northeastern Poland, (2) to identify the species and intraspecies diversity of the genus *Borrelia* in the study area, (3) to study the influence of conditions connected with the subregion, the biotope, and the year on tick density and the differences in the *Borrelia* species composition in *I. ricinus*, and (4) to determine the prevalence and intraspecific genetic diversity of *B. miyamotoi* in *I. ricinus* ticks in the study area.

## 2. Materials and Methods

### 2.1. Study Area and Tick Collection

Tick sampling was conducted at 15 sites near recreational areas, forest parking lots and picnic areas, and forest paths in six region districts of Warmia and Mazury in northeastern Poland (Figure 1, Table S1). The tick collection sites (surfaces of 400–500 m^2^) represented the western, central, and eastern parts of the region and two types of habitats: (a) forest landscapes (mature mixed and deciduous forests) and (b) ecotones (zones between grassy and forested areas such as paths near forest borders) (Table S1). At each site, questing *I. ricinus* ticks were collected during the springtime activity of ticks (April–June) of 2016 and 2017. Collections were performed twice per month in each year of the study during the daytime between 9 a.m. and 4 p.m. by two persons for at least 30 min using the standard flagging method. Ticks were not collected during and shortly after rainfall or on very sunny and hot days. Collected ticks were preserved in 70% ethanol. In the laboratory, specimens were identified by species, sex, and life stage using a taxonomic key [42] and were preserved individually (adults) or in pools (nymphs) at −80 °C for further molecular analysis.

### 2.2. DNA Extraction

The extraction of genomic DNA from ticks was carried out by universal kit Sherlock AX (A&A Biotechnology, Gdynia, Poland) according to the manufacturer’s instructions. DNA was isolated from individual specimens of adults and pools of five nymphs. Before DNA extraction, the ticks were air-dried for several minutes and then cut and crushed with a sterile scalpel. Extracted DNA was eluted in 40 μL of TE buffer and stored at −80 °C for further analysis.

### 2.3. Borrelia Spirochaete DNA Detection

The presence of *Borrelia* spirochaetes in tick genomic DNA isolates was confirmed by the amplification of different loci: (a) 16S rRNA gene (357 bp) with primers LDF/LDR [43], (b) outer surface protein A (*ospA*) gene (307 bp) with primers SL1/SL2 [44], and (c) the flagellin (*flaB*) gene with the primers BFL1/BFL2 [45,46] (422 bp) in conventional PCR and two sets of primers: outer—132f/905r (774 bp) and inner—220f/823r (604 bp) in nested PCR (nPCR) [47] (Table 1). All amplifications were performed with a total volume of 25 µL of PCR mixture containing 12.5 µL of 2 × PCR Master Mix Plus (0.1 U/µL of Taq polymerase supplied in a PCR buffer, 4 mM of MgCl2, and 0.5 mM of each dNTPs) (A&A Biotechnology, Gdynia, Poland), 0.5 µL of each primer (10 µM), 5 µL of template DNA (in nPCR—1 µL of template DNA or 1 µL of the outer PCR product), and an appropriate amount of sterile nuclease-free water. DNA isolated from a *B. afzelii*-positive *I. ricinus* tick (confirmed by sequencing in an earlier study) and nuclease-free water were run in each PCR as positive and negative controls, respectively. PCR amplicons were visualised on 1.5% agarose gels stained with Midori Green Stain (Nippon Genetics Europe, Düren, Germany) using GelDocXR (Bio-Rad, Hercules, CA, USA). Each DNA sample was considered *Borrelia*-positive when fragments of the 16S rRNA/*ospA* gene and fragment *flaB* gene were amplified.

Among *Borrelia*-positive samples in nPCR, *B. miyamotoi* DNA was also detected with specific primers BmF/BmR for the *flaB* marker [40]. All isolates positive for *B. miyamotoi* based on the *flaB* gene were confirmed by amplifying the fragment of the glycerophosphodiester phosphodiesterase (*glpQ*) gene (700bp) that is specific for relapsing fever *Borrelia* spp. [48] (Table 1).

### 2.4. Borrelia Species Identification by the PCR-RFLP Method

The restriction fragment length polymorphism (RFLP) method was used to identify *Borrelia* species. The characteristic patterns of DNA fragments were obtained by the digestion of amplicons of the *flaB* gene with a length of approximately 422 bp (amplified with BFL1/BFL2) and 604 bp (amplified with 220f/823r) by using the restriction enzymes Tsp 509I (*TasI*) and HpyF3I (*DdeI*) (ThermoFisher Scientific, Waltham, MA, USA), respectively. The digestions were performed according to the manufacturer’s instructions. Restriction fragments were separated on 3% agarose gel stained with Midori Green Stain (Nippon Genetics Europe, Düren, Germany) and visualised using GelDocXR (Bio-Rad, Hercules, CA, USA). The RFLP patterns obtained by using HpyF3I enzyme enabled the identification of nine Borrelia species: *B. afzelii*, *B. garinii/B. bavariensis*, *B. burgdorferi* sensu stricto (s.s.), *B. lusitaniae*, *B. valaisiana*, *B. bissetti*, *B. spielmanii,* and *B. miyamotoi,* which were included in the relapsing fever group of *Borrelia* [47,49]. The Tsp 509I enzyme allows for distinguishing *B. garinii* and *B. afzelii* from the group of other species of the *B. burgdorferi* s.l. complex: *B. burgdorferi* s.s., *B. lusitaniae*, *B. valaisiana*, *B. bissetti*, *B. spielmanii*, *B. finlandensis*, and *B. carolinensis* [46].

### 2.5. Borrelia Species Identification by DNA Sequencing

To confirm the typing of *Borrelia* species, 258 *flaB* genes and 9 *glpQ* gene-positive PCR products were purified using the CleanUp purification kit (A&A Biotechnology, Gdynia, Poland) according to the manufacturer’s protocol and sequenced bi-directionally with 220f/823r, BFL1/BFL2, BmF/BmR, or GLPQF/GLPQR primers (Macrogen Europe, Amsterdam, The Netherlands). The obtained nucleotide sequences were edited in BioEdit v. 7.2 software [50] (https://bioedit.software.informer.com, accessed on 20 March 2021) and compared with data registered in the GenBank database (http://www.ncbi.nih.gov/Genbank/index.html, accessed on 23 March 2021) using the BLAST-NCBI program (http://www.ncbi.nlm.nih.gov/BLAST/, accessed on 23 March 2021). Consensus sequences of the *Borrelia flaB* gene and *B. miyamotoi glpQ* gene were deposited in the GenBank database and registered under the accession numbers MW963151-MW963173. 

### 2.6. Phylogenetic Analysis

The phylogenetic analysis used *B. miyamotoi* and *B. bavariensis flaB* gene sequences that were obtained from the collected *I. ricinus* ticks and the most similar chosen reference sequences from GenBank. The phylogram was constructed using the Maximum Likelihood method based on the Kimura 2-parameter model. The topology of the phylogenetic tree was evaluated using the bootstrap method with 1000 replicates. Phylogenetic analysis was conducted using MEGA X software [51] (https://www.megasoftware.net, accessed on 30 April 2021).

### 2.7. Statistical Analysis

The density of *I. ricinus* ticks for each collection site was estimated by determining the number of ticks per 100 m^2^ for each flagging event. Differences in mean tick densities were evaluated by ANOVA with normal errors. The General Linear Model (GLM) of One Variable was used to test the main effects of Year (2016, 2017), Region (West, Central, East), and Habitat (forest landscape, ecotone) on the density of *I. ricinus* ticks (nymphs, females, males, total). A chi-square test or Fisher’s exact test (when the expected frequency was < 5 in at least one of the cells of the contingency table) and 95% confidence intervals (95% CI) were used to compare the differences in the prevalence of *Borrelia* spirochaetes in the tested population and the distribution of *Borrelia* species in infected ticks between developmental stages of ticks, years, regions, and habitats. Borrelia infection in nymphs (tested in pools of five) was presented as the Minimum Infection Rate (MIR) and estimated as the ratio of the number of positive pools to the total number of tested samples, assuming only one infected tick specimen in a positive pool. 

The analysis was conducted using the software package SPSS version 27.0 for Windows (SPSS Inc., Chicago, IL, USA). In all analyses, *p*-values below 0.05 were considered statistically significant. 

## 3. Results

### 3.1. Tick Density

**Figure 2:**
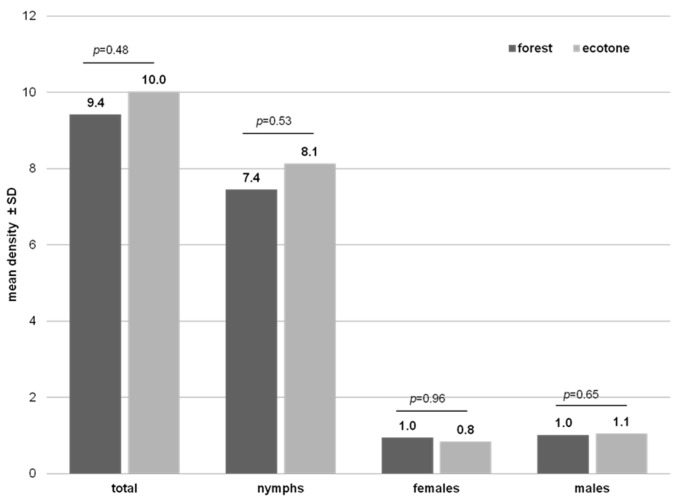
Comparison of *Ixodes ricinus* mean density (ticks per 100 m^2^) according to habitats in northeastern Poland (ANOVA, GLM, *p* < 0.05).

In 2016 and 2017, during the springtime activity of ticks, a total of 4334 *I. ricinus* were collected, which was comprised of 3473 nymphs and 861 adults (399 females, 462 males). The overall mean density was 9.7 ticks per 100 m^2^ (Table 2). There were no differences between the years, regions, or habitats of the study (Table 2, Figure 2, Table S2); only the interaction between year and region had significant effects on the total tick density (*Year × Region*: F_2,89_ = 3.5, *p* = 0.036). In 2016, the *I. ricinus* mean density was significantly higher in the western subregion (11.7 ticks/100 m^2^) in comparison to the central (8.9 ticks/100 m^2^) subregion (Table 2, Table S2).

During the study, the highest mean density was noted for nymphs (7.8/100 m^2^). The year of study had a significant effect on the mean nymph density (*Year*: F_1,89_ = 11.7, *p* = 0.001). The presence of nymphs was 8.8 and 6.8 ticks per 100 m^2^ in 2016 and 2017, respectively. The mean nymph density also differed between regions (*Region*: F_2,89_ = 5.7, *p* = 0.005). In the western subregion, nymph density was significantly higher (9.2/100 m^2^) than in the central (7.6 ticks/100 m^2^) and eastern (6.6 ticks/100 m^2^) subregions (Table 2, Table S2).

The mean density of adults was similar, i.e., 0.9 and 1.0 ticks per 100 m^2^, for females and males, respectively. The mean density of females was significantly different between regions (*Region*: F_2,89_ = 3.8, *p* = 0.027) (Table 2, Table S2). In males, the year of study had a significant effect on the mean density (*Year*: F_1,89_ = 6.0, *p* = 0.016) (Table 2, Table S2).

### 3.2. Prevalence of Borrelia Spirochaetes

For the presence of *Borrelia* DNA, a total of 4281 *I. ricinus* ticks were tested, including 861 specimens of adults (399 females, 462 males) and 3420 nymphs (tested in 684 pools, 53 nymphs did not form complete pools from a given flagging event and collection site). In 2016–2017, the overall natural tick infection rate was 8.1% (345/4281) and differed significantly between the developmental stages of ticks (χ^2^ = 166.96, *p* < 0.001) (Table 3). *Borrelia* DNA was detected in 22.6% (90/399) of the females and 14.3% (66/462) of the males. Among nymphs, the MIR was 5.5% (189/3420). There were significant differences in *Borrelia* spp. infection in *I. ricinus* between the year of the study (χ^2^ = 10.24, *p* < 0.001) (Table 3). *Borrelia* DNA was confirmed in 6.8% and 9.5% of the tested DNA samples in 2016 and 2017, respectively. The lowest *Borrelia* spp. prevalence in *I. ricinus* ticks was recorded in the western subregions of Warmia and Mazury (5.7%, 89/1559) compared to the central (8.7%, 118/1351) and eastern (10.1%, 138/1371) subregions (χ^2^ = 19.90, *p* < 0.001) (Table 3). The type of habitat did not affect the *Borrelia* infection rate (χ^2^ = 2.96, *p* = 0.085) (Table 3). In the population of ticks from forest areas, the level of infection was 8.8% (172/1945) and 7.4% (173/2336) in the ecotones. 

### 3.3. Borrelia Species Distribution

**Figure 3:**
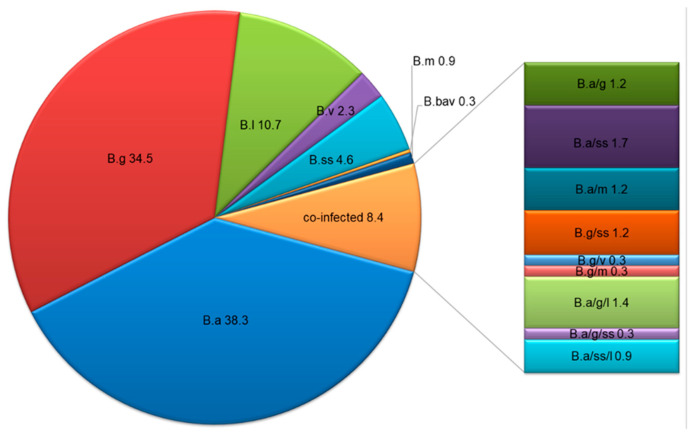
Species distribution (%) among *Borrelia*-positive *Ixodes ricinus* ticks, northeastern Poland (abbreviation: B.a—*B. afzelii*, B.g—*B. garinii*, B.ss—*B. burgdorferi* s.s., B.v—*B. valaisiana*, B.l—*B. lusitaniae*, B.bav—*B. bavariensis*, B.m—*B. miyamotoi*).

Among the *Borrelia*-positive samples, six species from the *B. burgdorferi* s.l. group (*B. afzelii*, *B. garinii*, *B. burgdorferi* s.s., *B. valaisiana*, *B. lusitaniae*, and *B. bavariensis*) and *B. miyamotoi* (from the group of spirochetes causing TBRF) were identified by PCR-RFLP and/or sequencing (Figure 3). In 91.6% (316/345, 95% CI: 89–95%) of samples, *Borrelia* species occurred as mono-infections. Species typing revealed the domination of single infections that are pathogenic for humans: *B. afzelii* (38.3%, 132/345), *B. garinii* (34.5%, 119/345), and *B. lusitaniae* (10.7%, 37/345). The remaining species occurred as single infections in about 8% of *Borrelia-*positive samples (Figure 3).

**Figure 4:**
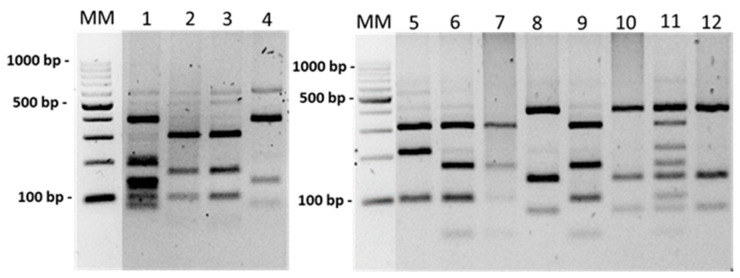
HpyF3I restriction patterns of the amplified fragment of *flaB* gene (604 bp) of *Borrelia* species. MM—DNA marker. Samples 1—*B. garinii/B. valaisiana* coinfection, 2, 3, 6, 7, 9—*B. afzelii*, 4, 8, 10, 12—*B. garinii*, 5—*B. lusitaniae*, 11—B*. afzelii/B. garinii/B. lusitaniae* coinfection.

Co-infections were identified in 8.4% (29/345, 95% CI: 5–11%) of the tested DNA samples. Two different *Borrelia* species were detected in 5.9% (20/345), and three were detected in 2.6% (9/345) of positive samples. Double infections included *B. afzelii*/*B. garinii*, *B. afzelii*/*B. burgdorferi* s.s., *B. garinii*/*B. burgdorferi* s.s., and *B. garinii*/*B. valaisiana*, and triple infections included *B. afzelii*/*B. garinii*/*B. lusitaniae*, *B. afzelii*/*B. garinii*/*B. burgdorferi* s.s., and *B. afzelii*/*B. burgdorferi* s.s./*B. lusitaniae* (Figure 3 and Figure 4).

*B. miyamotoi* was identified as a mono-infection in three *Borrelia-*positive samples (3/345, 0.9%) and in four *Borrelia-*positive samples as a co-infection with *B. afzelii,* as well as in one with *B. garinii* (5/345, 1.5%) (Figure 3, Table 4). All co-infections of *B. miyamotoi* occurred in DNA samples isolated from pools of nymphs.

The distribution of *Borrelia* species showed significant differences between *I. ricinus* stages (χ^2^ = 56.57, *p* = 0.002) (Table 4). Mono-infections were the most common in females and males, with 92.2% and 93.9%, respectively. Adult ticks were more frequently infected with *B. afzelii* (42.2% for females, 40.9% for males). In nymphs, *B. garinii* dominated (MIR: 78/189, 41.3%) (Table 4). About 62% (18/29) of co-infections were detected in nymphs. However, the analysis of co-infections in nymphs is not justified because the DNA was extracted from pooled tick samples. In adult *I. ricinus,* co-infections were more frequent in females than males (Table 4). Females carried only double infections of *Borrelia* spp., and none were infected with three species. In males, double and triple infections of *Borrelia* species appeared in equal proportions (Table 4).

The year of study and the biotope did not affect the *Borrelia* species composition (Table 4). Significant differences were only found between subregions (χ^2^ = 46.96, *p* < 0.05) (Table 4). *B. garinii* dominated in the western subregion of Warmia and Mazury and constituted 42.7% (38/89) of all positive samples in this region. In the central subregion, *B. afzelii* was most frequently identified (50.8%, 60/118), while in the eastern subregion, both species were found in comparable proportions, with 34.1% (47/138) and 32.6% (45/138) of positive samples, respectively. *B. miyamotoi* as a mono-infection occurred with equal frequency in each subregion, while it occurred as a co-infection with *B. afzelii* in the western subregion of Warmia and Mazury. Co-infection of *B. miyamotoi* and *B. garinii* was identified in the eastern subregion (Table 4).

### 3.4. Genetic Diversity in flaB Gene in Borrelia Species

To confirm *Borrelia* species identification based on RFLP of the *flaB* gene, 58 (44%) PCR amplicons were typed as *B. afzelii,* and all 184 were typed as other *Borrelia* species from a total of 316 mono-infected samples that were sequenced. All obtained chromatograms were checked manually, and 114 sequences with good quality obtained in nPCRs (~604 bp) were used for genetic diversity analysis. 

Overall, 21 variants of the *flaB* gene were identified (Table S3). The most polymorphic species was *B. garinii*. Among the 37 sequenced samples, 11 *flaB* gene variants were recognised. Nine of them were previously identified in *I. ricinus* ticks questing and feeding on the hosts in Poland and other European countries and in the tissues of wild mice of the genus *Apodemus*, which were considered to be reservoir species for *Borrelia* spp. (Table S3). Two variants, i.e., BgV4 (n = 4) and BgV10 (n = 1) (Table S3), were unique and displayed 99.6% and 99.8% nucleotide identity to sequences detected in *I. ricinus* from different parts of Poland and the Czech Republic (GenBank: MK604255, KF990320, JN828685). 

In *B. lusitaniae,* the second-most polymorphic species among 23 sequenced samples, four *flaB* gene variants were identified (Table S3). Two of them (BlV1 and BlV3) showed 100% nucleotide identity with sequences obtained from *I. ricinus* from Poland, Romania, and Turkey, as well as from the spleens of *Apodemus* mice from Poland. Variants BlV2 and BlV4 were not deposited previously in GenBank and showed 99.8% and 99.6% identity to sequences detected in *I. ricinus* from central (GenBank: MF150075) and western (GenBank: KF422804) Poland and Romania (GenBank: MW272741) (Table S3).

Only two variants were recognised among 40 *flaB* gene sequences of *B. afzelii*. Variant BaV1, which was detected in 38 sequenced samples, showed 100% identity to the strains BO23 (GenBank: CP018262) and K78 (GenBank: CP009058) that were detected in symptomatic patients with borreliosis in Germany and Austria (Table S3). This pathogenic variant also occurred in *I. ricinus* from another part of Poland (GenBank: MK604271). The second variant of *B. afzelii* BaV2 was identified in only two sequenced samples and displayed the highest similarity with sequences from questing *I. ricinus* from Poland and the Czech Republic (GenBank: KR782215, KF422856, JN828691).

No diversity of the *flaB* gene was detected among sequenced samples of *B. burgdorferi* s.s. and *B. valaisiana* (Table S3). The *B. burgdorferi* s.s. variant (n = 5) showed 100% nucleotide identity to sequences that occurred in patients from the Czech Republic (GenBank: FJ231335) and in ticks from Poland and Germany. The variant of *B. valaisiana* (n = 5) was also previously identified in *I. ricinus* from Poland and Lithuania and *I. persulcatus* in Siberia (Russia) (Table S3). 

**Figure 5:**
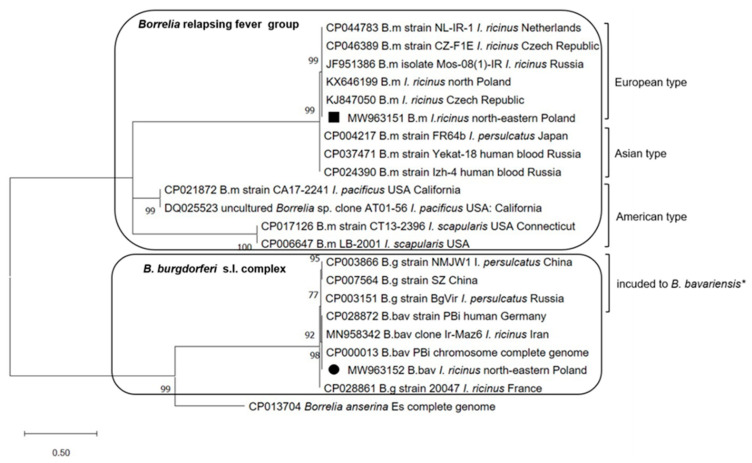
The molecular relationship between *Borrelia miyamotoi* and *Borrelia bavariensis* is based on the sequences of the *flaB* gene identified in the study. The phylogram was constructed using the Maximum Likelihood method and Kimura 2-parameter method as a distance method. The percentage of replicate trees in which the associated taxa are clustered together in the bootstrap test (1000 replicates) is shown next to the branches. The tree is drawn to scale, with branch lengths measured in the number of base substitutions per site. The analyses and phylogram construction were conducted in MEGA X software (https://www.megasoftware.net (accessed on 30 April 2021)). The sequences obtained in the study are labelled with black symbols. Abbreviation: B.m—*B. miyamotoi*, B.g—*B. garinii*, B.bav—*B. bavariensis*. * according to Becker et al. (2020) [52].

The single *B. bavariensis* sequence detected in an *I. ricinus* male from the central subregion of Warmia and Mazury was identical with sequences of Pbi strain isolated from a human sample in Germany (GenBank: CP028872) and a *I. ricinus* tick from Iran (GenBank: MN958342) (Figure 5, Table S3).

In *B. miyamotoi,* positive ticks from all three sequences of the *flaB* gene fragments (from mono-infected isolates) (GenBank: MW963151) were monomorphic and showed 100% nucleotide identity to the sequences of *B. miyamotoi* strains obtained from naturally infected *I. ricinus* ticks from the Czech Republic, the Netherlands, northern Poland, and Russia (European type) (Figure 5). Belonging to the European type of *B. miyamotoi,* a lack of polymorphism was also confirmed based on the sequence analysis of a fragment of the *glpQ* gene detected in nine isolates from mono- and co-infected samples (GenBank: MW963173).

## 4. Discussion

The authors’ long-term monitoring of tick prevalence in northeastern Poland revealed that *I. ricinus*, a vector of human-pathogenic *Borrelia* spirochaetes, is the most abundant tick (with mean density 9.7 ticks per 100 m^2^) in northeastern Poland. In the central and eastern subregions of Warmia and Mazury (especially in open landscapes), the presence of *Dermacentor reticulatus* ticks was also confirmed, but with a much lower mean density (1.9 and 2.7 ticks per 100 m^2^ in natural and urban areas, respectively) [53]. In the studied population of *I. ricinus* ticks, a higher mean density of nymphs (7.8 ticks per 100 m^2^) than adult ticks was noted. The higher density of nymphs compared to adult ticks is consistent with the fact that most reported tick bites on humans are from nymphs [54,55,56,57]. Nymphs are therefore considered the most important life stage involved in transmitting *Borrelia* spirochetes to humans.

The density of *I. ricinus* in natural biotopes of this region is almost five times higher than in green recreational areas in Olsztyn, the capital of the Warmia and Mazury region [41]. Such disproportions in the average tick population density between urban and natural ecotypes have been confirmed in other areas [40,58,59,60]. Many studies have indicated that tick density in a given area depends on the local properties of the habitat, which affects the differences between regions, biotopes, and years of the study [40,60,61]. However, in northeastern Poland, tick density seems to be constant with a comparable level in subsequent years, subregions, and biotopes, both in forest landscapes and ecotones. This is probably due to the relative homogeneity of the area in terms of the shape of the surface and natural features and the mosaic structure with complexes of forests, lakes, peat bogs, used meadows, pastures, agricultural land, and relatively low human interference [62]. This ensures the optimal structure of vegetation and microclimate for ticks at the studied sites and access to mammalian hosts (rodents, deer) [63,64], which affects the reproduction of ticks and the maintenance of their population. 

The landscape of the Warmia and Mazury region, with a high degree of forest cover and many lakes, allows and encourages residents to engage in outdoor activities (walking, picking berries and mushrooms, etc.) and is conducive to the development of tourism [65]. Such patterns of human behaviour bring people into contact with habitats populated by ticks and increase the risk of tick-borne infections [65,66,67].

The revealed average frequency (8.1%) of *Borrelia* spirochetes in *I. ricinus* in northeastern Poland is in the range of 0.25–12.4%, which was recorded in other regions of the country [68,69]. It is also in concordance with results from northeastern Poland over the last twenty years [38,70,71,72]. The overall level of infection in the *I. ricinus* population in northeastern Poland was identical to that noted in the current study, despite the use by Stańczak et al. (1999) [71] of a less sensitive method (indirect immunofluorescence assay) of spirochaete detection. The proportions of infected *I. ricinus* adults and nymphs examined by Pawełczyk and Siński (2004) [39] two decades ago were also similar. It is assumed that the risk of being bitten by an infected tick has not radically changed. The relatively constant prevalence of *B. burgdorferi* s.l. in the questing ticks over the past two decades has been confirmed by two meta-analyses [11,73] and a revisited study conducted in Hanover (Germany) [74] and in Ireland [75]. A higher prevalence of *Borrelia* spp. in *I. ricinus* in northeastern Poland was recorded to date only in the green areas of Olsztyn—the largest city of Warmia and Mazury—both in questing ticks (27%) [41] and feeding on dogs (35.7%) [76]. A lower level of *Borrelia* infection was noted in I*. ricinus* feeding on deer (5.4%) from northeastern Poland, which is associated with a confirmed elimination of *Borrelia* infection in ticks feeding on roe deer and other wild ungulates, possibly due to the bacteriolytic complement pathway in ungulate blood [64]. 

The level of *Borrelia* spp. infection in *I. ricinus* in northeastern Poland is more than two times lower than the calculated projection in a meta-analysis (19.3%) based on the results of studies from 2010–2016 on ticks from Central European countries [11]. This is undoubtedly due to the limitation of the current study resulting from the pooling of nymphs and using the minimum infection rate (MIR) to estimate the prevalence of *Borrelia* spp. and assuming that one nymph in the pool is infected. This reduced the overall mean incidence of spirochetes in *I. ricinus* ticks. The level of infection in adult ticks individually examined by us was similar to the infection rate reported by Strnad et al. (2017) [11] (18% vs 21.6%, respectively), but in nymphs tested in pools (MIR 5.5%) it was three times lower than the level of infection in nymphs tested individually (16.7%). This is also confirmed by a higher level of co-infection in nymphs than in adults, which have a much greater chance of acquiring *Borrelia* spirochetes than nymphs. However, the current data confirm the general trend that the level of infection in adult ticks is much higher than in nymphs [11,68]. In contrast to *I. ricinus* density, the *Borrelia* spirochete infection rate was significantly different between the years of the study and the subregions. The most *Borrelia* spp. infected region was the eastern subregion of Warmia and Mazury, where the incidence of LB among the inhabitants is over 20% higher than in the other subregions (Figure 1). The results from previous studies [77,78] indicate differences in the prevalence of *Borrelia* spp. in ticks from habitats (e.g., fragmented forest plots, continuous forests, ecotones) with a different biodiversity of vertebrates (hosts of ticks). However, the risk of acquiring LB in northeastern Poland seems to be similar in both study habitats forest and ecotones.

In the population of *I. ricinus* ticks in northeastern Poland, the richness of the spirochaete species was recorded. To date, among the twelve species of the *B. burgdorferi* s.l. complex identified in Europe [55], six were detected in northeastern Poland. Most of them have been confirmed as pathogenic to humans. In concordance with the meta-analysis results of data from 2010–2016 concerning the species composition of *Borrelia* spirochaetes in ticks from 23 European countries [11], *B. afzelii* and *B. garinii* species were also predominant in *I. ricinus* in this study. Those two species were identified in 75% of ticks. Despite the highest frequency, both species show extremely different genetic diversity within the partial sequence of the *flaB* gene; *B. garinii* was the most polymorphic of the identified species. Among 21 of the recognised variants in the *flaB* gene, over 50% occurred in *B. garinii*. In contrast, *B. afzelii* was the least diverse species, which was represented by only two variants of the *flaB* marker. A greater diversity of *flaB* markers within *B. garinii* was also recognised by Kowalec et al. (2017) [40] in natural ecotypes in eastern Poland, which can be explained by the high rates of avian host migration with which *B. garinii* is associated [68].

In *I. ricinus* of the Warmia and Mazury region, the current study did not confirm the occurrence of the pathogenic *B. spielmanii* previously detected in *I. ricinus* in Poland in the forested city areas of Warsaw [40] or in *I. ricinus* removed from humans [54]. However, *B. bavariensis* (formerly *B. garinii* OspA serotype 4) [79] was identified in *I. ricinus* males from the central subregion of Warmia and Mazury. As far as it is known, this species has not been previously recorded in ticks in Poland [68] and is not considered a public health issue in Poland, although antibodies against the p18 protein of *B. bavariensis* are present in 5% of foresters and 3% of farmers in southeastern Poland [80]. Recently, *B. bavariensis* has become of great interest due its isolation from LB patients in Europe, although detection in questing *I. ricinus* is very rare in Europe [11,81,82,83,84] and reports are mostly from Asia [52]. 

The genetic diversity of the *Borrelia* spirochetes is involved with differences in the clinical presentation and invasiveness of LB in humans. *B. afzelii* is most frequently associated with skin manifestations (*erythrema migrans*, *acrodermatitis chronica atrophicans,* or *borrelial lymphocytoma*), while *B. garinii* and *B. bavariensis* are most often associated with neuroborreliosis. In contrast, the pathogenicity *of B. burgdorferi* s.s. is related to neuroborreliosis and arthritis symptoms. Some species, such as *B. lusitaniae*, have only occasionally been associated with human disease [32,68]. The current finding of *B. miyamotoi* in *I. ricinus* in northeastern Poland as a mono-infection and co-infection with *B. afzelii* and *B. garinii* may also change the clinical picture of LB and its severity and make diagnosis and treatment difficult. It has been observed that *B. miyamotoi* is constantly circulating in European populations of questing *I. ricinus*, although its prevalence is low and ranges between 0.2% and 8.9%, depending on the region and the developmental stage of ticks [20]. A relatively high *B. miyamotoi* prevalence was detected in *I. ricinus* ticks removed from humans in Poland (8.4%) [54] and Germany (7.3%) [55]. In Poland, *B. miyamotoi* was also identified in *I. ricinus* in recreational areas in Szczecin [47] and Warsaw [40], and in natural habitats of Lower Silesia [85] and eastern Poland [40], with a prevalence ranging from 0.5–3.9%. In the Warmia and Mazury region, *B. miyamotoi* was identified in questing *I. ricinus* in green urban areas [41] and ticks feeding on deer [64]. Despite suggesting the presence of a genetically specific Polish strain of *B. miyamotoi* [40], the genetic analysis in the current study revealed a lack of polymorphism in the *flaB* gene and *glpQ* gene sequences and full nucleotide identity to the European type of *B. miyamotoi*. Despite the low prevalence of *B. miyamotoi* in *I. ricinus* in northeastern Poland, the authors strongly agree with the conclusion [40,54,55] that *B. miyamotoi* disease (BMD) should not be underestimated. The number of confirmed symptomatic and asymptomatic cases of BMD in Europe is steadily increasing and has been diagnosed so far in 50 patients, including one patient in Poland [20,26]. Therefore, physicians should be aware of *B. miyamotoi* infections among patients with unspecific feverish illness or with neurological symptoms that do not meet the criteria for neuroborreliosis (anti-*Borrelia* antibodies detected only in serum) [86].

Due to the lack of effective and commercially available human vaccines against LB, its control is limited to reducing the risk of contamination in the environment and encouraging the public to take preventive measures to avoid exposure to ticks [66,87]. Although human behaviour may affect the risk of tick exposure, space-time estimates of tick density and pathogen prevalence are necessary for these measures to be effective. It seems that research on local prevalence has a very limited value in terms of epidemiological risk assessment [11]. However, the integration of data from studies based on similar methodologies allows for the analysis (models, meta-analysis) of spatial and temporal changes and trends in determining human tick-borne disease incidence, including LB [11,35,66,73,88,89]. Moreover, for a disease of growing public health importance, and which is likely to affect increasing numbers of people, local government and healthcare professionals need to understand the current burden in their region [89,90,91,92]. 

## 5. Conclusions

The density of ticks in northeastern Poland is constant, regardless of the subregion, habitat, or year of study. Nevertheless, the risk of developing LB is high due to the prevalence and richness of the *Borrelia* species. Most of the *Borrelia* species identified in the *I. ricinus* population in northeastern Poland are human pathogens. An analysis of their frequency suggests a high probability of skin symptoms of LB caused by *B. afzelii* infection and cases of neuroborreliosis caused by *B. garinii*. Co-infection with several species of *Borrelia* spp. or infection/co-infection with *B. miyamotoi* may change the clinical picture of LB. Therefore, physicians should be aware of this and consider it when diagnosing patients suspected of having a tick-borne disease.

## Figures and Tables

**Table 1:** Primers and conditions of annealing step of PCRs used for detection of *Borrelia* spp.

Locus	Primer Name	Primer Sequence 5′-3′	Product Size [bp]	Reference	Annealing Step in PCR
***Borrelia* spp.**					
16S rRNA	LDF	ATGCACACTTGGTGTTAACTA	357	[43]	53 °C/30 s
	LDR	GACTTATCACCGGCAGTCTTA			
*ospA*	SL1	AATAGGTCTAATAATAGCCTTAATAGC	307	[44]	65 °C/90 s
	SL2	CTAGTGTTTTGCCATCTTCTTTGAAAA			
*flaB*	BFL1	GCTCAATATAACCAAATGCACATG	422	[45,46]	58 °C/45 s
	BFL2	CAAGTCTATTTTGGAAAGCACCTAA			
	132f	TGGTATGGGAGTTCTGG	774	[47]	52 °C/30 s
	905r	TCTGTCATTGTAGCATCTTT			
	220f	CAGACAACAGAGGGAAAT	604		54 °C/20 s
	823r	TCAAGTCTATTTTGGAAAGCACC			
***B. miyamotoi***					
*flaB*	BmF	AACTTGCTGTTCAGTCTGGT	424	[40]	54 °C/20 s
	BmR	TTAACTCCACCTTGAACTGG			
*glpQ*	forward	ATGGGTTCAAACAAAAAGTCACC	700	[48]	53 °C/30 s
	reverse	CCAGGGTCCAATTTCATCAGAATATTGTGCAAC			

**Table 2:** Mean *Ixodes ricinus* density (ticks per 100 m^2^) according to subregion and year of the study in northeastern Poland.

	Year	Subregion			Mean ± SD
		West	Central	East	
**Nymphs**	2016	10.6 ± 3.25 ^a^	7.6 ± 2.37 ^b^	8.3 ± 2.91 ^a,b^	8.8 ± 3.08 ^a^
	2017	7.7 ± 2.84 ^a^	7.6 ± 2.84 ^a^	4.9 ± 2.10 ^b^	6.8 ± 2.87 ^b^
	**Mean**	9.2 ± 3.33 ^a^	7.6 ± 2.56 ^b^	6.6 ± 3.03 ^b^	7.8 ± 3.14
**Females**	2016	0.5 ± 0.45 ^a^	0.6 ± 0.78 ^a^	1.1 ± 1.31 ^a^	0.8 ± 0.94 ^a^
	2017	0.7 ± 0.65 ^a^	1.1 ± 0.77 ^a^	1.3 ± 0.86 ^a^	1.0 ± 0.79 ^a^
	**Mean**	0.6 ± 0.56 ^a^	0.8 ± 0.79 ^a,b^	1.2 ± 1.10 ^b^	0.9 ± 0.87
**Males**	2016	0.6 ± 0.53 ^a^	0.7 ± 0.84 ^a^	1.0 ± 1.18 ^a^	0.8 ± 0.89 ^a^
	2017	0.9 ± 1.07 ^a^	1.5 ± 1.21 ^a^	1.5 ± 1.12 ^a^	1.3 ± 1.14 ^b^
	**Mean**	0.8 ± 0.85 ^a^	1.1 ± 1.09 ^a^	1.3 ± 1.15 ^a^	1.0 ± 1.05
**Total**	2016	11.7 ± 3.25 ^a^	8.9 ± 2.49 ^b^	10.5 ± 3.39 ^a,b^	10.4 ± 3.22 ^a^
	2017	9.4 ± 3.22 ^a^	10.2 ± 2.89 ^a^	7.7 ± 2.64 ^a^	9.1 ± 3.04 ^a^
	**Mean**	10.9 ± 4.40 ^a^	9.5 ± 2.73 ^a^	9.1 ± 3.30 ^a^	9.7 ± 3.2

^a,b^—different letters mean significant differences (post-hoc Bonferroni test, ANOVA, GLM).

**Table 3:** Prevalence of *Borrelia* spp. in *Ixodes ricinus* ticks by life stage, year, subregion, and habitat in northeastern Poland.

		No. of Tested Ticks	*Borrelia*-Positive n/% * (95% CI)	*p*-Value **
**Stage**	Nymphs	3420	189/5.5 ^a^ (4.8–6.3)	<0.001
	Females	399	90/22.6 ^b^ (18.4–26.7)	
	Males	462	66/14.3 ^c^ (11.1–17.5)	
**Year**	2016	2313	158/6.8 ^a^ (5.8–7.9)	<0.001
	2017	1968	187/9.5 ^b^ (8.2–10.8)	
**Subregion**	West	1559	89/5.7 ^a^ (4.6–6.9)	<0.001
	Central	1351	118/8.7 ^b^ (7.2–10.2)	
	East	1371	138/10.1 ^b^ (8.5–11.7)	
**Habitat**	Forest	1945	172/8.8 ^a^ (7.6–10.1)	0.085
	Ecotone	2336	173/7.4 ^a^ (6.3–8.5)	
**Total**		4281	345/8.1 (7.2–8.9)	

*—for nymphs Minimum Infection Rate (MIR) is given (5 nymphs per isolate); **—chi2 test, *p* < 0.05; ^a,b,c^—different letters mean significant differences (post -hoc Bonferroni test).

**Table 4:** *Borrelia* species distribution in positive *Ixodes. ricinus* ticks by life stage, year, subregion, and habitat in northeastern Poland.

		Stage (n/%)			Year (n/%)		Subregion (n/%)			Habitat (n/%)	
		F	M	N *	2016	2017	West	Central	East	Forest	Ecotone
**mono-infection**	B.a	38 ^a^/42.2	27 ^a^/40.9	67 ^a^/35.4	65 ^a^/41.1	67 ^a^/35.8	27_b_/30.3	60 ^a^/50.8	45_b_/32.6	65 ^a^/37.8	67 ^a^/38.7
	B.g	27_a.b_/30.0	14_b_/21.2	78 ^a^/41.3	51 ^a^/32.3	68 ^a^/36.4	38 ^a^/42.7	34 ^a^/28.8	47 ^a^/34.1	60 ^a^/34.9	59 ^a^/34.1
	B.l	14 ^a^/15.6	13 ^a^/19.7	10 ^b^/5.3	17 ^a^/10.8	20 ^a^/10.7	8 ^a,b^/9.0	4 ^a^/3.4	25_b_/18.1	14 ^a^/8.1	23 ^a^/13.3
	B.v	0 ^a^/0.0	1 ^a^/1.5	7 ^a^/3.7	3 ^a^/1.9	5 ^a^/2.7	2 ^a^/2.2	4 ^a^/3.4	2 ^a^/1.4	2 ^a^/1.2	6 ^a^/3.5
	B.ss	4 ^a^/4.4	5 ^a^/7.6	7 ^a^/3.7	8 ^a^/5.1	8 ^a^/4.3	3 ^a^/3.4	7 ^a^/5.9	6 ^a^/4.3	13 ^a^/7.6	3 ^b^/1.7
	B.bav	0 ^a^/0.0	1 ^a^/1.5	0 ^a^/0.0	0 ^a^/0.0	1 ^a^/0.5	0 ^a^/0.0	1 ^a^/0.8	0 ^a^/0.0	0 ^a^/0.0	1 ^a^/0.6
	B.m	0 ^a^/0.0	1 ^a^/1.5	2 ^a^/1.1	1 ^a^/0.6	2 ^a^/1.1	1 ^a^/1.1	1 ^a^/0.8	1 ^a^/0.7	1 ^a^/0.6	2 ^a^/1.2
	**Subtotal**	83 ^a^/92.2	62 ^a^/93.9	171 ^a^/90.5	145 ^a^/91.8	171 ^a^/91.4	79 ^a^/88.8	111 ^a^/94.1	126 ^a^/91.3	155 ^a^/90.1	161 ^a^/93.1
**co-infection**	B.a/B.g	1 ^a^/1.1	0 ^a^/0.0	3 ^a^/1.6	1 ^a^/0.6	3 ^a^/1.6	0 ^a^/0.0	1 ^a^/0.8	3 ^a^/2.2	2 ^a^/1.2	2 ^a^/1.2
	B.a/B.ss	4 ^a^/4.4	2 ^a^/3.0	0 ^b^/0.0	0 ^a^/0.0	6 ^b^/3.2	1 ^a^/1.1	2 ^a^/1.7	3 ^a^/2.2	4 ^a^/2.3	2 ^a^/1.2
	B.a/B.m	0 ^a^/0.0	0 ^a^/0.0	4 ^a^/2.1	3 ^a^/1.9	1 ^a^/0.5	3 ^a^/3.4	0 ^a^/0.0	1 ^a^/0.7	2 ^a^/1.2	2 ^a^/1.2
	B.g/B.ss	1 ^a^/1.1	0 ^a^/0.0	3 ^a^/1.6	2 ^a^/1.3	2 ^a^/1.1	3 ^a^/3.4	1 ^a^/0.8	0 ^a^/0.0	3 ^a^/1.7	1 ^a^/0.6
	B.g/B.v	1 ^a^/1.1	0 ^a^/0.0	0 ^a^/0.0	1 ^a^/0.6	0 ^a^/0.0	0 ^a^/0.0	0 ^a^/0.0	1 ^a^/0.7	1 ^a^/0.6	0 ^a^/0.0
	B.g/B.m	0 ^a^/0.0	0 ^a^/0.0	1 ^a^/0.5	0 ^a^/0.0	1 ^a^/0.5	0 ^a^/0.0	0 ^a^/0.0	1 ^a^/0.7	1 ^a^/0.6	0 ^a^/0.0
	B.a/B.g/B.l	0 ^a^/0.0	0_a_/0.0	5 ^a^/2.6	3 ^a^/1.9	2 ^a^/1.1	1 ^a^/1.1	2 ^a^/1.7	2 ^a^/1.4	3 ^a^/1.7	2 ^a^/1.2
	B.a/B.g/B.ss	0 ^a^/0.0	1 ^a^/1.5	0 ^a^/0.0	0 ^a^/0.0	1 ^a^/0.5	1 ^a^/1.1	0 ^a^/0.0	0 ^a^/0.0	0 ^a^/0.0	1 ^a^/0.6
	B.a/B.ss/B.l	0 ^a^/0.0	1 ^a^/1.5	2 ^a^/1.1	3 ^a^/1.9	0 ^a^/0.0	1 ^a^/1.1	1 ^a^/0.8	1 ^a^/0.7	1 ^a^/0.6	2 ^a^/1.2
	**Subtotal**	7 ^a^/7.8	4 ^a^/6.1	18 ^a^/9.5	13 ^a^/8.2	16 ^a^/8.6	10 ^a^/11.2	7 ^a^/5.9	12 ^a^/8.7	17 ^a^/9.9	12 ^a^/6.9
	*p*-value **	0.002			0.355		0.025			0.318	

*—for nymphs Minimum Infection Rate (MIR) is given (5 nymphs per isolate); **—chi2 test, *p* < 0.05 (for Borrelia species distribution); ^a,b^—different letters mean significant differences (post-hoc Bonferroni test); (abbreviation: F—females, M—males, N—nymphs; B.a—*B. afzelii*, B.g—B. garinii, B.ss—*B. burgdorferi* s.s., B.v—*B. valaisiana*, B.l—*B. lusitaniae*, B.bav—*B. bavariensis*, B.m—*B. miyamotoi*).

## Data Availability

Not applicable.

## Supplementary Materials

The following supporting information can be downloaded at: https://www.mdpi.com/article/10.3390/ijerph19127378/s1, Table S1: Characteristics of *I. ricinus* tick collection localities in north-eastern Poland; Table S2: Statistical table of ANOVA (GLM) analysis *of I. ricinus* tick density in north-eastern Poland; Table S3: Variants of *flaB* gene in spirochaetes species from *B. burgdorferi* s.l complex identified in questing *I. ricinus* in north-eastern Poland.
