# Supplementary material for: Abundance of Ixodes ricinus Ticks (Acari: Ixodidae) and the Diversity of Borrelia Species in Northeastern Poland
Source: Int J Environ Res Public Health. 2022 Jun 16;19(12):7378. doi: 10.3390/ijerph19127378 (PMC9223791; doi:10.3390/ijerph19127378)
Supplement: Supplementary file 1 [file ijerph-19-07378-s001.zip › ijerph-1744114-supplementary.pdf]

International Journal of
Environmental Research and Public Health

MDPI

Table S1. Characteristics of *I. ricinus* tick collection localities in north-eastern Poland.

Region	Province	Locality	Geographical coordinates	Habitats
West	Iława	Szałkowo	53°39'52.0"N 19°37'03.8"E	Ecotone (grassy path/forest)
		Sąpy	53°42'30.9"N 19°40'52.8"E	Ecotone (grassy path/forest)
		Wielowieś	53°46'39.5"N 19°44'58"E	Forest landscape
	Ostróda	Zawady Małe	53°41'39.2"N 20°05'25.1"E	Forest landscape
		Samborowo	53°40'22.3"N 19°47'47.7"E	Ecotone (grassy path/forest)
Central	Olsztyn	Kudypy	53°45'58.9"N 20°22'47.4"E	Ecotone (grassy path/forest)
		Zazdrość	53°41'45.6"N 20°31'21.3"E	Forest landscape
	Szczytno	Czarny Piec	53°32'35.6"N 20°37'38.8"E	Ecotone (grassy path/forest)
		Warchały	53°32'15.6"N 20°49'14.6"E	Forest landscape
		Leleszki	53o37′02.6"N 20°43′17.3"E	Ecotone (grassy path/forest
East	Mrągowo	Wierzbowo	53°48'24.0"N 21°19'25.6"E	Ecotone (grassy path/forest)
		Piecki	53°48'01.7"N 21°19'33.1"E	Forest landscape
		Sorkwity	53°50'19.0"N 21°06'22.6"E	Forest landscape
	Pisz	Krutyń	53°42'09.0"N 21°26'21.4"E	Forest landscape
		Wygryny	53°40'15.8"N 21°32'17.1"E	Ecotone (grassy path/forest)

Table S2. Statistical table of ANOVA (GLM) analysis of *I. ricinus* tick density in north-eastern Poland.

Population	General Linear Model (GLM)	Effects and effect interactions statistics
**Total (females + males + nymphs)**	**Year × Region × Habitat**	Main effect of Year: $F_{1, 89}$ = 3.6, p = 0.061<br>Main effect of Region: $F_{2, 89}$ = 1.3, p = 0.281<br>Main effect of Habitat: $F_{1, 89}$ = 0.5, p = 0.484<br>Effect interaction of Year × Habitat: $F_{1, 89}$ = 0.0, p = 0.958<br>Effect interaction of Year × Region: $F_{2, 89}$ = 3.5, p = 0.036*<br>Effect interaction of Habitat x Region : $F_{2, 89}$ = 0.1, p=0.862<br>Effect interaction of Year × Region x Habitat: $F_{2, 89}$ = 0.4, p=0.695
**Nymphs**	**Year × Region × Habitat**	Main effect of Year: $F_{1, 89}$ = 11.7, p = 0.001*<br>Main effect of Region: $F_{2, 89}$ = 5.7, p = 0.005*<br>Main effect of Habitat: $F_{1, 89}$ = 0.4, p = 0.529<br>Effect interaction of Year × Habitat: $F_{1, 89}$ = 0.1, p = 0.762<br>Effect interaction of Year × Region: $F_{2, 89}$ = 2.8, p = 0.068

		Effect interaction of Habitat x Region : $F_{2, 89}$ = 0.3, p=0.752<br>Effect interaction of Year × Region x Habitat: $F_{2, 89}$ = 0.8, p=0.452
**Females**	**Year × Region × Habitat**	Main effect of Year: $F_{1, 89}$ = 2.0, p = 0.159<br>Main effect of Region: $F_{2, 89}$ = 3.8, p = 0.027*<br>Main effect of Habitat: $F_{1, 89}$ = 0.0, p = 0.955<br>Effect interaction of Year × Habitat: $F_{1, 89}$ = 0.1, p = 0.707<br>Effect interaction of Year × Region: $F_{2, 89}$ = 0.4, p = 0.605<br>Effect interaction of Habitat x Region : $F_{2, 89}$ = 0.2, p=0.784<br>Effect interaction of Year × Region x Habitat: $F_{2, 89}$ = 0.0, p=0.981
**Males**	**Year × Region × Habitat**	Main effect of Year: $F_{1, 89}$ = 6.0, p = 0.016*<br>Main effect of Region: $F_{2, 89}$ = 2.5, p = 0.092<br>Main effect of Habitat: $F_{1, 89}$ = 0.2, p = 0.647<br>Effect interaction of Year × Habitat: $F_{1, 89}$ = 0.1, p = 0.709<br>Effect interaction of Year × Region: $F_{2, 89}$ = 0.4, p = 0.656<br>Effect interaction of Habitat x Region : $F_{2, 89}$ = 2.8, p=0.066<br>Effect interaction of Year × Region x Habitat: $F_{2, 89}$ = 0.5, p=0.586

* statistically significant - $p<0.05$.

Table S3. Variants of *flaB* gene in spirochaetes species from *B. burgdorferi* s.l complex identified in questing *I. ricinus* in north-eastern Poland.

**Species**	**Variant**	**Isolate (n)**	**Accession number in GenBank**	**Similarity in GenBank**			
				**Reference in GenBank**	**Identity (%)**	**Host**	**Country**
B. bavariensis	BbavV1	1	MW963152	CP028872 (strain Pbi)	100	human	Germany
				MN958342		*I. ricinus*	Iran
B. burgdorferi	BbV1	5	MW963153	MF150052	100	*I. ricinus*	Poland
				CP001205 (strainZS7)		tick	Germany
				FJ231335		human	Czech Republic
B. valaisiana	BvV1	5	MW963154	MF150077	100	*I. ricinus*	Poland
				MT813126			Lithuania
				CP009117 (strain Tom4006)		*I. persulcatus*	Siberia
B. lusitaniae	BlV1	12	MW963155	MF150075	100	*I. ricinus*	central Poland

				KF422804			western Poland
				MW272741			Romania
	BlV2	5	MW963156	MF150075	99.8	*I. ricinus*	central Poland
				KF422804			western Poland
				MW272741			Romania
	BlV3	5	MW963157	KR782242	100	*I. ricinus*	Poland
				AB091809			Turkey
				KF894062		*Apodemus agrarius* (spleen)	Poland
	BlV4	1	MW963158	MF150075	99.6	*I. ricinus*	central Poland
				KF422804			western Poland
				MW272741			Romania
B. garinii	BgV1	6	MW963159	MK604254	100	*I. ricinus*	Poland
				KF990320			S-W Poland
				JN828685			Czech Republic
	BgV2	5	MW963160	KF918606	100	*I. ricinus* fed on *Vulpes vulpes*	Poland
				MK604257		*I. ricinus*	
				KF836510			S-W Poland
	BgV3	5	MW963161	KF422775	100	*I. ricinus* fed on *Nyctereutes procyonoides*	Poland
				KF836512			S-W Poland
				HM345905		*I. ricinus*	western Poland
	BgV4	4	MW963162	MK604255	99.6	*I.ricinus*	Poland
				KF990320			S-W Poland
				JN828685			Czech Republic
	BgV5	3	MW963163	MK604261	100	*I. ricinus*	Poland
				KF894053		*Apodemus flavicollis* (spleen)	
				KF422816		*I. ricinus* fed on *Dama dama*	
	BgV6	3	MW963164	MK604253	100	*I. ricinus*	Poland
				AB091814			Turkey

				KJ577820		*I. ricinus* fed on *Myotis daubentonii*	Poland
	BgV7	3	MW963165	KF422783<br>KF894058<br>HM345902	100	I. ricinus<br>*Apodemus flavicollis* (spleen)<br>*I. ricinus* fed on *Turdus philomelos*	Poland
	BgV8	4	MW963166	KX646198<br>DQ650336<br>KF894055	100	*I. ricinus*<br>*Apodemus agrarius* (spleen)	Poland
	BgV9	2	MW963167	MK604263<br>KF990322<br>MN958341	100	*I. ricinus*	Poland<br>western Poland<br>Iran
	BgV10	1	MW963168	MK604255<br>KF990320<br>JN828685	99.8	*I. ricinus*	Poland<br>S-W Poland<br>Czech Republic
	BgV11	1	MW963169	CP028861 (strain 200047)<br>KR782222<br>KF894061	100	*I. ricinus* fed on bird<br>*I. ricinus*<br>*Apodemus agrarius* (spleen)	France<br>Poland<br>Poland
B. afzelii	BaV1	38	MW963171	CP018262 (strain BO23)<br>CP009058 (strain K78)<br>MK604271	100	human skin<br>*I. ricinus*	Germany<br>Austria<br>Poland
	BaV2	2	MW963172	KR782215<br>KF422856<br>JN828691	100	*I. ricinus*	N-W Poland<br>Poland<br>Czech Republic
